# Atrial Fibrillation Prediction Model Following Aortic Valve Replacement Surgery

**DOI:** 10.3390/jcdd12020052

**Published:** 2025-01-31

**Authors:** Nora Knez, Tomislav Kopjar, Tomislav Tokic, Hrvoje Gasparovic

**Affiliations:** 1 Institute of Emergency Medicine of the City of Zagreb, 10000 Zagreb, Croatia; noraknez6@gmail.com; 2 Department of Cardiac Surgery, University Hospital Center Zagreb, 10000 Zagreb, Croatia; tokic.klc@hotmail.com (T.T.); hgasparovic@gmail.com (H.G.); 3 School of Medicine, University of Zagreb, 10000 Zagreb, Croatia

**Keywords:** aortic valve replacement, postoperative atrial fibrillation, prediction model

## Abstract

(1) Background: Postoperative atrial fibrillation (POAF) is the most common complication following cardiac surgery. It leads to increased perioperative morbidity and costs. Our study aimed to determine the incidence of new-onset POAF in patients undergoing isolated aortic valve replacement (AVR) and develop a multivariate model to identify its predictors. (2) Methods: We conducted a retrospective study including all consecutive patients who underwent isolated AVR at our institution between January 2010 and December 2022. Patients younger than 18, with a history of atrial fibrillation, previous cardiac surgery, or those who underwent concomitant procedures were excluded. Patients were dichotomized into POAF and No POAF groups. Multivariate logistic regression with backward elimination was utilized for predictive modeling. (3) Results: This study included 1108 patients, of which 297 (27%) developed POAF. The final multivariate model identified age, larger valve size, cardiopulmonary bypass time, delayed sternal closure, ventilation time, and intensive care unit stay as predictors of POAF. The model exhibited fair predictive ability (AUC = 0.678, *p* < 0.001), with the Hosmer–Lemeshow test confirming good model fit (*p* = 0.655). The overall correct classification percentage was 65.6%. (4) Conclusions: A POAF prediction model offers personalized risk estimates, allowing for tailored management strategies with the potential to enhance patient outcomes and optimize healthcare costs.

## 1. Introduction

Postoperative atrial fibrillation (POAF) is the most common complication following cardiac surgery [1]. The incidence of POAF after cardiac surgery varies greatly between studies [2]. It occurs in approximately up to 50% of all patients undergoing aortic valve replacement surgery [3]. Likewise, the incidence of new-onset atrial fibrillation following transcatheter aortic valve implantation (TAVI) is also high and associated with a marked increase in incident length of stay, stroke, and in-hospital mortality [3]. The differences in pathologies that are more associated with atrial fibrillation than others might be responsible for the widespread incidence of POAF after cardiac surgery [4]. However, significant variability in POAF definition across different research studies might also contribute to this variability. Some groups report POAF episodes without specifying duration or treatment necessity, while others employ duration-based definitions with arbitrary cut-offs [5]. Additionally, certain studies only include POAF episodes requiring treatment or intervention [6].

Postoperative atrial fibrillation has been associated with numerous detrimental consequences for patients following cardiac surgery [7]. Increased perioperative morbidity, mortality, and costs have all been associated with POAF [8]. It has been widely recognized as an independent predictor of early and long-term cardiovascular complications, including stroke, thromboembolism, cardiac arrest, the need for permanent pacemaker implantation, and increased hospital length of stay [9]. The underlying pathophysiological pathways contributing to the incidence of POAF following cardiac surgery remain unclear and persist as an active area of research. The mechanism of POAF development is multifactorial, involving preoperative, surgical, anesthetic, and postoperative factors [10]. Cardiac surgery induces physiological disturbances that may be superimposed on the susceptible atrial substrate, making the atrium more vulnerable to the induction and maintenance of atrial fibrillation, including vasoplegia, inflammation, catecholamine release, altered autonomic nervous system activity, fluid shifts, and myocardial ischemia.

In this study, we aimed to determine the incidence of POAF in patients undergoing isolated aortic valve replacement (AVR) surgery and to design a multivariate model that identifies clinical and procedural predictors of POAF development. A prediction model for POAF offers personalized risk estimates, allowing for tailored management strategies that have the potential to enhance patient outcomes and optimize healthcare costs.

## 2. Materials and Methods

### 2.1. Study Design

A retrospective cohort study was conducted to investigate the predictors of POAF. All consecutive patients who underwent isolated AVR from January 2010 to December 2022 at the University Hospital Center Zagreb were eligible for inclusion. The only inclusion criterion was the first-time isolated AVR. Patients younger than 18, those with a history of atrial fibrillation, or those with previous cardiac surgery were excluded from the study. Additionally, we excluded patients with concomitant procedures. Demographic, clinical, and laboratory data were retrospectively collected from individual medical records in a computerized database. Patients were monitored continuously during the entire postoperative hospital stay using telemetry until discharge and were dichotomized regarding new-onset POAF occurrence into POAF and No POAF groups.

### 2.2. Outcomes

The primary outcome of this study was to determine the incidence of POAF after isolated AVR. The secondary outcome was to identify preoperative, intraoperative, and postoperative risk factors associated with POAF development. POAF was defined as any AF detected on telemetry, lasting for 10 min or more, and confirmed with a 12-lead electrocardiogram (ECG) during the postoperative period before hospital discharge. Any such event would qualify the patients into the POAF group. Neither the treatment of POAF nor the success of treatment had any influence on the decision to group the patient into the POAF or No POAF group.

### 2.3. Aortic Valve Replacement

Patients receiving preoperative beta blockers were given their normal dose on the morning of surgery. The patients received diazepam and morphine 30 min before the induction of anesthesia. Aortic valve replacement was performed following our standardized institutional protocol for isolated AVR surgery. Either biological or mechanical valves were implanted mainly based on patients’ age. However, the final valve choice was based on patients’ preferences after an informed interview with the operating surgeon. The conventional surgical approach to the aortic valve is via a midline sternotomy. A minimally invasive AVR is performed via a partial upper sternotomy with a “J” extension into the third or fourth intercostal space. The choice to perform minimally invasive AVR was based on the surgeon’s preference. In conventional and minimally invasive AVR procedures, we utilize central cardiopulmonary bypass with cannulation of the ascending aorta and right atrium performed on an arrested heart. Myocardial protection consisted of antegrade cardioplegia. Systemic heparinization was used, followed by full reversal with protamine after decannulation. Vacuum-assisted venous drainage was used ubiquitously, and flooding the operative field with CO_2_ facilitated deairing. Postoperatively, all patients are admitted to a dedicated cardiac intensive care unit (ICU). Patients are typically transferred to an intermediate care unit on the second postoperative day. Early postoperative care focuses on fluid management, electrolyte balance, atrial fibrillation prophylaxis, respiratory physiotherapy, and early mobilization.

### 2.4. Detection and Management of Atrial Fibrillation

All patients were continuously monitored with telemetry until hospital discharge. A 12-lead ECG was obtained on the day before discharge. Any clinical suspicion of arrhythmia during the hospital stay was followed by a 12-lead ECG and reinstitution of telemetry monitoring.

Although there was no standardized prophylactic protocol for POAF prevention that was uniformly applied across the cohort, the practice of atrial fibrillation prevention has not changed during the study period. It was based on the best medical judgment of the treating physicians for all the patients. Patients were routinely started on beta blockers on postoperative day one or once inotropic support was suspended. Amiodarone was not routinely administered. Electrolyte levels were measured on a daily basis, and throughout their hospital stay, magnesium and potassium supplements were administered to maintain high normal levels of these electrolytes. The aim was to optimize volume substitution to preserve adequate preload without causing depletion. Anemia was corrected usually with two doses of erythrocyte concentrates if the hemoglobin levels were <80 g/dL and hematocrit was <22%. Atrial fibrillation was treated with amiodarone, sometimes in conjunction with electrical cardioversion. In cases of atrial flutter with poor rate control, digoxin was used in conjunction with beta-blockers and amiodarone.

### 2.5. Categorization of Risk Factors

In this study, we aimed to analyze the risk factors associated with POAF development in patients undergoing isolated AVR. The variables were categorized into preoperative, intraoperative, and postoperative risk factors allowing a deeper understanding of the multifactorial nature of POAF by highlighting the interplay between patient-specific characteristics, procedural factors, and postoperative recovery dynamics. Preoperative metrics included demographic and clinical variables such as age, sex, body mass index (BMI), comorbidities (e.g., hypertension and coronary artery disease), EuroSCORE II, and preoperative left ventricular ejection fraction. These factors were selected based on strong evidence in the literature demonstrating their roles as established predictors of POAF. Intraoperative metrics encompassed factors like valve type (bioprosthetic vs. mechanical), valve size, cardiopulmonary bypass duration, and the use of a minimally invasive approach. These variables were included for their potential to influence intraoperative stress, inflammation, and hemodynamic alterations, which are key contributors to arrhythmogenesis. Postoperative metrics focused on the duration of mechanical ventilation, need for reintubation, ICU stay, delayed sternal closure, and wound infection. These were chosen for their strong association with early recovery dynamics and postoperative complications, which may predispose patients to arrhythmias during the immediate recovery period.

### 2.6. Statistical Analysis

Statistical analysis was performed using IBM SPSS Statistics 29.0.2.0. Two-sided *p*-values of <0.05 were considered statistically significant. The normality of distribution was tested using the Kolmogorov–Smirnov test. Continuous variables were reported as the mean +/− standard deviation or median with interquartile range, as appropriate. Variables with normal distributions were compared with independent samples t-tests and those with non-normal distributions were compared with Mann–Whitney tests. Dichotomous variables were presented with frequencies and percentages. Pearson’s χ2 test was used to compare the frequencies between the groups unless the number of events was lower than 5, in which case, Fisher’s exact test was used.

Multivariate logistic regression analysis was utilized to design a prediction model for identifying patients at risk of POAF. Clinically significant independent variables in univariate analysis with a *p*-value of <0.2 were entered into the binary logistic regression model to determine their independent effects on POAF. The model was constructed using a backward elimination approach, wherein variables with the least statistical significance (*p* > 0.05) were iteratively removed from the model while ensuring the inclusion of clinically relevant variables. Odds ratios (ORs) with 95% confidence intervals (CIs) were reported. After conducting multivariate logistic regression analysis, the Hosmer–Lemeshow test was used to assess the model’s goodness of fit. A non-significant *p*-value (*p* > 0.05) indicated that the predicted probabilities aligned well with the observed data, confirming the model’s calibration. A classification table was generated to evaluate the model’s predictive accuracy, assessing sensitivity, specificity, and overall accuracy using a cut-off value of 0.3. The predictive value was determined by the area under the curve (AUC) in the receiver operating characteristic (ROC) analysis.

## 3. Results

**Figure 1:**
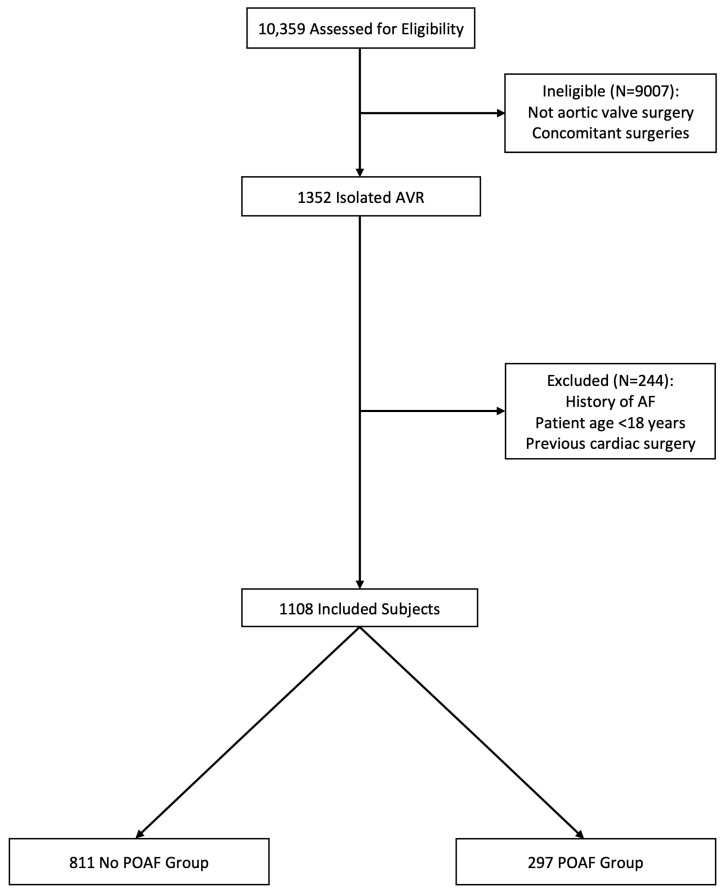
Study flowchart. The study flowchart provides information on numbers with reasons for patient enrollment, allocation, and analysis. AF: atrial fibrillation; AVR: aortic valve replacement; POAF: postoperative atrial fibrillation.

The overall number of adult patients undergoing isolated AVR in our institution during the study period was 1352. Of those, 244 patients were excluded and the final study population included 1108 patients (Figure 1).

Of the 1108 patients in the final study population, 652 (59%) were male and 456 (41%) were female. The median age was 68 (60–74) years. Demographic data with clinical profiles are summarized in Table 1. Postoperative atrial fibrillation was observed in 297 subjects, which accounted for 27% of the entire cohort. Patients in the POAF groups were older and had higher EuroSCORE II surgical risk profiles. Other basic demographic characteristics, such as sex and BMI, were comparable between the study groups. Hypertension and non-obstructive coronary artery disease were more common in the POAF group. The bicuspid aortic valve was more common in the No POAF group.

Intraoperative variables are presented in Table 2. It was revealed that the bioprosthetic valves were used in 724 cases, accounting for 65% of the entire cohort. Of the 724 bioprosthetic valves, 51 (7%) were rapid deployment valves. Mechanical valves were less commonly used, accounting for 384 cases (35%). The POAF group had more bioprosthetic valves, while the No POAF group had more mechanical valves. Cardiopulmonary bypass and aortic cross-clamp times were longer in the No POAF group. Valve sizes were similar between groups, as well as the use of a minimally invasive approach.

Hospital outcomes are presented in Table 3. Delayed sternal closure and reintubation were more frequent in the POAF group. The duration of days spent in the ICU and mechanical ventilation times were higher in the POAF group. The frequency of other postoperative complications was comparable between the groups, including mortality (2 [0.7%] vs. 10 [1.2%], *p* = 0.531).

Of all the variables, 17 clinically significant independent variables from the univariate analysis with a *p*-value of <0.2 were entered into the logistic regression model to determine their independent effects. For the final multivariate analysis model, a group of independent variables that had a significant relationship with POAF are reported in Table 4. Out of the variables included in the final model, older age, larger valve size, shorter cardiopulmonary bypass time, delayed sternal closure, shorter ventilation time, and longer ICU stay were independent predictors of POAF.

**Figure 2:**
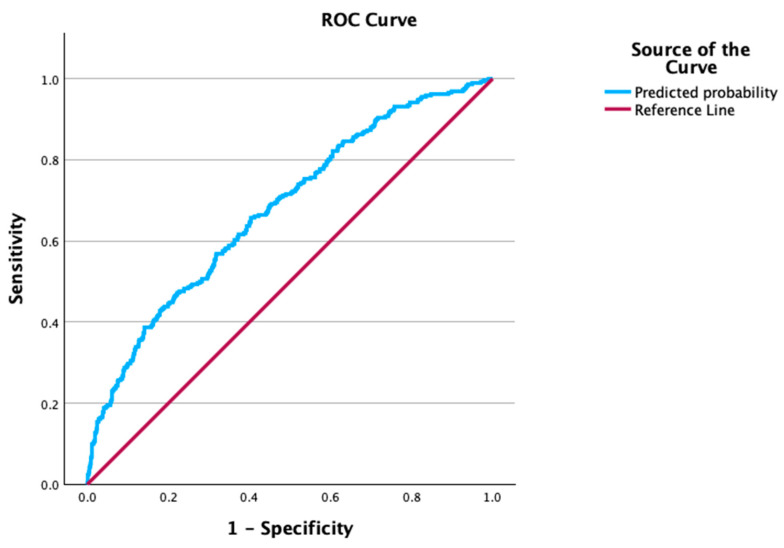
Receiver operating characteristic curve. The curve depicts a point score as a prediction of postoperative atrial fibrillation among patients undergoing isolated aortic valve replacement surgery.

The ROC curve analysis shown in Figure 2 shows a fair predictive ability of the model (AUC = 0.678, *p* < 0.001, 95% CI = 0.642–0.714). The Hosmer–Lemeshow test yielded a non-significant *p*-value for the final model (*p* = 0.655), indicating a good model fit. The classification table (Table 5) demonstrated an accuracy of 65.6%, with a specificity of 68.9% and a sensitivity of 56.6%, at a cut-off value of 0.3.

## 4. Discussion

Our study found that the incidence rate of POAF was 27% among adult patients undergoing first-time isolated AVR surgery. This is consistent with previous findings reported in the literature [3,11,12]. Despite improvements in surgical techniques and perioperative prevention strategies, POAF remains the most common complication following cardiac surgery [1,2]. In a large contemporary population-based retrospective study, the incidence of new-onset atrial fibrillation is observed in approximately 50% of cases during hospitalizations for both TAVI and AVR [3]. The POAF prevalence rate highlights the importance of identifying potential risk factors to prevent increased morbidity, mortality, and treatment costs [8,13]. Managing POAF remains a significant clinical challenge, encompassing rhythm and rate control, as well as the prevention of thromboembolic events.

Our final multivariate logistic regression analysis revealed several variables significantly associated with the development of POAF after isolated AVR. It identified older age, larger valve size, delayed sternal closure, and longer ICU stay as independently associated with POAF. It also identified shorter cardiopulmonary bypass and mechanical ventilation times as predictors of POAF. While the overall accuracy of the model was 65.6%, its specificity (68.9%) demonstrates strong performance in identifying patients without POAF. However, the sensitivity (56.6%) highlights a limitation in detecting POAF cases. Future model improvements should enhance sensitivity while maintaining acceptable specificity to balance predictive accuracy. The Hosmer–Lemeshow test results indicated a good model fit, suggesting that the model is reliable and can be used for POAF prediction in patients undergoing isolated AVR.

Age has consistently been identified and widely accepted as an independent predictor of new-onset POAF development after cardiac surgery [6,13,14]. Aging leads to fibrotic processes that result in structural and electrophysiological changes in the atria. Among other studies, we confirmed this in a study previously published by our group [15]. In an arrhythmogenic environment, triggers such as cardiac surgery may result in the development of arrhythmias [12,13]. In the univariate analysis, we found a strong and highly significant association between age and the development of POAF. In the multivariate analysis, advanced age remained a significant predictor of POAF, underscoring its importance as a key risk factor for this complication in patients undergoing isolated AVR. In the univariate analysis, mechanical ventilation duration was significantly higher in the POAF group. The OR for mechanical ventilation in the multivariate analysis was slightly smaller than 1 (0.99) with a *p*-value of 0.025, indicating that mechanical ventilation was negatively associated with POAF after controlling for other factors. Although mechanical ventilation duration was inversely associated with POAF, the length of ICU stay was positively associated with POAF and had a high statistical significance with a *p*-value less than 0.001. Some of these findings were unexpected, given that previous studies have found prolonged mechanical ventilation to be a risk factor for POAF [14,16,17]. Although some factors, such as age (OR = 1.04) and ventilation time (OR = 0.99), were statistically significant predictors of POAF, their odds ratios close to 1 indicate modest practical effects. For instance, each additional year of age is associated with only a 4% increase in the risk of POAF, while each additional hour of ventilation is associated with a 1% decrease in risk. While these effects are minor in isolation, they remain clinically relevant when combined with other predictors in the multivariate model, offering a more comprehensive understanding of POAF risk. Future research should explore interactions among predictors to elucidate better their combined impact on clinical outcomes. Many factors identified as contributors to POAF are closely linked to prolonged cardiopulmonary bypass time, highlighting the interdependence of procedural and recovery dynamics. This relationship is further compounded by advanced age, a well-established risk factor for both prolonged cardiopulmonary bypass time and POAF. Contrary to previous findings, cardiopulmonary bypass duration demonstrated an OR of 0.99, indicating a slight negative association with POAF. This discrepancy may reflect differences in patient characteristics or procedural factors specific to this cohort. The duration of the cardiopulmonary bypass has been previously associated with POAF in a study published by our group, including patients subjected to first-time isolated coronary bypass surgery [14]. Furthermore, while statistically significant, postoperative metrics such as ICU stay are less practical for early risk stratification, as they become apparent only after surgery. Developing a predictive model based solely on preoperative and intraoperative variables could offer a valuable tool for identifying high-risk patients before surgery.

Patients with impaired renal function may have higher levels of inflammatory markers and electrolyte derangements, which have been implicated in the pathogenesis of POAF [8,12]. In the univariate analysis, chronic kidney disease requiring dialysis was found to be borderline significant for its association with POAF (*p* = 0.064). Dialysis was one of the clinically significant independent variables included in the multivariate analysis. However, it did not reveal itself as an independent predictor of POAF. On the other hand, delayed sternal closure and increased valve size were independent predictors of new-onset POAF after AVR. It is difficult to explain how an increase in valve size corroborates an increase in POAF risk. Delayed sternal closure is likely associated with increased surgical risk, which can easily explain the association. There are multiple possible explanations for the occurrence of new-onset AF after AVR. It can be due to a combination of patient substrate and surgery-specific precipitating factors. The patient-related substrate is mirrored in our study through the higher odds of POAF with increasing age and surgical risk profile revealed by increased EuroSCORE II. However, EuroSCORE II was not found to be an independent risk factor for POAF development in our cohort. This finding underscores the complexity of POAF risk, suggesting that EuroSCORE II reflects overall surgical risk but lacks specificity for direct POAF prediction. There are many potential precipitating triggers in the perioperative period. A hypersensitive and alert state could be driven by a combination of pain and myocardial trauma, among other factors. Local inflammation triggered by AVR has previously been linked to the new-onset POAF occurrence [18]. The surgical trauma to the myocardium and the sympathovagal fibers may be the drivers of this inflammation [19]. Additionally, our study highlights the intricate interplay of factors that collectively contribute to the arrhythmogenic substrate in this population. These findings validate existing knowledge while emphasizing the importance of a multifactorial approach to POAF prediction and management tailored to the unique profiles of isolated AVR patients.

One of the factors associated with POAF that has been previously explored is the BMI. In our study, BMI was similar in both study groups, and it did not show any association with POAF. The direction of the effect in previous studies suggests that higher BMI may increase the risk for POAF development. This has not been consistently reported in all previous studies. Some studies have reported that this does not account for the entire BMI spectrum. Studies have highlighted the importance of a non-linear relationship between excessive body weight and adverse outcomes following CABG [20,21]. The exact mechanism underlying the association between BMI and POAF is unclear. Studies have shown that BMI is an LA size determinant, and LA enlargement is a strong POAF predictor [22]. Additionally, it is hypothesized that obesity-related metabolic changes and chronic low-grade inflammation may play a role [23,24]. These findings highlight the importance of identifying patients at risk for POAF and implementing targeted interventions to prevent this complication.

The higher proportion of bioprosthetic valves in the POAF group and mechanical valves in the No POAF group reflects differences in patient selection. Bioprosthetic valves are often chosen for older, comorbid patients who are inherently at higher risk for POAF due to age and frailty. In contrast, mechanical valves are typically used in younger patients, who experience fewer complications and shorter ICU stays, reducing POAF risk. This highlights the interplay between valve type, patient characteristics, and POAF, warranting further research into the underlying mechanisms.

Several limitations of the study must be considered. Firstly, this is a retrospective study based on medical records, which may affect the completeness and quality of the data. It is crucial to acknowledge the potential for data collection bias inherent in the retrospective study design, which may introduce limitations to the validity of the findings. The data were collected from a single center, which limits the number of patients and may affect the generalizability of the results. Furthermore, this study did not consider other potential risk factors for POAF occurrence, such as serum electrolyte levels, inflammatory parameters, and left atrial dimensions. While our research focused on in-hospital detection of POAF, this period primarily captures transient arrhythmias driven by inflammation or surgical stress, which may be resolved after discharge. Persistent POAF, which carries distinct long-term risks and management implications, was not assessed. Monitoring POAF after discharge presents a challenge, particularly with continuous rhythm assessment, which often requires invasive techniques. However, non-invasive approaches, such as follow-up protocols involving regular hospital visits and periodic ECG monitoring, offer practical and feasible alternatives for evaluating cardiac rhythm, ensuring that patients remain in sinus rhythm, or identifying recurrences of atrial fibrillation. Future studies should incorporate extended follow-up periods and advanced monitoring methods to differentiate transient from persistent POAF, enabling more reliable insights for targeted management strategies. Additionally, postoperative metrics in our predictive model have limited utility for preoperative risk stratification. Future models should prioritize preoperative and intraoperative variables to enhance the early identification of high-risk patients. These models should not only be applied to training but also to testing datasets to validate their performance. The absence of such a validation procedure in the present study could lead to model and training data overfitting, potentially limiting its application in other populations. While logistic regression was chosen for its simplicity, interpretability, and clinical relevance, alternative machine learning techniques, such as decision trees, random forests, and gradient boosting, may improve predictive accuracy by capturing complex, non-linear relationships among variables. These approaches are particularly suited to imbalanced datasets and can leverage ensemble methods to enhance sensitivity for minority classes. Future research should explore these methods to evaluate their potential to outperform traditional regression models in predictive accuracy and clinical utility.

## 5. Conclusions

The multivariate prediction model developed in this study offers a practical and reliable tool for identifying patients at high risk of developing new-onset POAF following isolated AVR. By leveraging this model, healthcare providers can stratify POAF risk more effectively, enabling targeted interventions, improved resource allocation, and better patient outcomes while potentially reducing healthcare costs. Our findings highlight advanced age as a significant predictor of POAF, emphasizing its importance as a key risk factor in this population. While our study underscores the multifactorial nature of POAF, future prospective studies are essential to evaluate the efficacy of prophylactic interventions, such as left atrial appendage closure or pulmonary vein isolation, in reducing POAF-related morbidity and mortality. Although our findings do not directly test these strategies, their potential to reduce the need for long-term anticoagulation therapy and alleviate the burden of POAF warrants further investigation. Additionally, future research should explore advanced machine learning techniques to capture better complex, non-linear relationships among predictors and address the class imbalance challenges. Extended follow-up periods are also critical to differentiate transient from persistent POAF, offering a deeper understanding of its long-term clinical implications and enabling more effective management strategies.

## Figures and Tables

**Table 1:** Demographic and clinical profiles of subjects undergoing isolated aortic valve replacement surgery.

	All Subjects (n = 1108)	No POAF (n = 811)	POAF (n = 297)	*p*-Value
Age (years)	68 (60–74)	67 (59–73)	71 (65–76)	<0.001
Female	456 (41)	322 (40)	134 (45)	0.105
Body mass index (kg/m^2^)	29 (25–32)	28 (25–32)	29 (25–32)	0.459
Ejection fraction (%)	60 (50–65)	60 (50–65)	60 (50–65)	0.207
Hemoglobin (g/L)	133 (121–144)	134 (121–144)	133 (122–142)	0.395
Aortic stenosis	1010 (91)	735 (91)	276 (93)	0.23
Bicuspid aortic valve	243 (22)	193 (24)	50 (17)	0.013
Endocarditis	29 (3)	26 (3)	3 (1)	0.054
Coronary artery disease	190 (17)	126 (16)	64 (22)	0.019
Hypertension	879 (79)	630 (78)	249 (84)	0.025
Diabetes mellitus	281 (25)	201 (25)	80 (27)	0.466
Dyslipidemia	584 (53)	422 (52)	162 (55)	0.458
Smoking	282 (25)	212 (26)	70 (24)	0.384
Chronic obstructive pulmonary disease	94 (8)	66 (8)	28 (9)	0.495
Dialysis	9 (0.8)	4 (0.5)	5 (2)	0.064
Previous solid organ transplantation	3 (0.3)	2 (0.2)	1 (0.3)	1.000
Peripheral vascular disease	39 (4)	27 (3)	12 (4)	0.569
Previous myocardial infarction	65 (6)	42 (5)	23 (8)	0.107
Stroke	112 (10)	83 (10)	29 (10)	0.818
EuroSCORE II (%)	2.3 (1.5–3.5)	2.2 (1.5–3.3)	2.7 (1.8–4.3)	<0.001

Values are medians with interquartile ranges or frequencies with percentages.

**Table 2:** Intraoperative characteristics of the study subjects.

	All Subjects (n = 1108)	No POAF (n = 811)	POAF (n = 297)	*p*-Value
Bioprosthetic valve	724 (65)	494 (61)	230 (77)	<0.001
Valve size (mm)	23 (21–24)	23 (21–24)	23 (21–25)	0.188
Minimally invasive approach	326 (29)	236 (29)	90 (30)	0.697
Cardiopulmonary bypass (min)	98 (81–121)	100 (82–123)	93 (78–114)	0.002
Aortic cross-clamp (min)	68 (55–85)	69 (56–88)	65 (53–80)	0.003

Values are medians with interquartile ranges or frequencies with percentages.

**Table 3:** Comparison of in-hospital outcomes between the study groups.

	All Subjects (n = 1108)	No POAF (n = 811)	POAF (n = 297)	*p*-Value
Mechanical circulatory support	7 (0.6)	5 (0.6)	2 (0.7)	1.000
Delayed sternal closure	8 (0.7)	3 (0.4)	5 (2)	0.036
Revision due to bleeding	28 (3)	23 (3)	5 (2)	0.278
Reintubation	10 (0.9)	4 (0.5)	6 (2)	0.027
Ventilation (hours)	7 (6–10)	7 (5–10)	8 (6–12)	<0.001
Intensive care unit stay (days)	2 (1–2)	2 (1–2)	2 (1–3)	<0.001
Need for pacemaker	24 (2)	16 (2)	8 (3)	0.467
Stroke	12 (1.1)	9 (1.1)	3 (1)	1.000
Sternal wound infection	29 (3)	18 (2)	11 (4)	0.173

Values are medians with interquartile ranges or frequencies with percentages. Bolded variables were included in the multivariate logistic regression model.

**Table 4:** Variables included in the final model of multivariate analysis.

	Odds Ratio	95% Confidence Interval	*p*-Value
Age	1.04	1.03–1.06	<0.001
Female	1.37	0.97–1.93	0.075
Endocarditis	0.34	0.09–1.31	0.118
Coronary artery disease	1.41	0.98–2.03	0.065
Valve size (mm)	1.12	1.03–1.22	0.010
Cardiopulmonary bypass (min)	0.99	0.99–0.99	0.002
Delayed sternal closure	41.32	3.56–480.13	0.003
Ventilation (h)	0.99	0.99–0.99	0.025
Intensive care unit (days)	1.22	1.11–1.36	<0.001

R^2^ = 0.12, *p* < 0.001.

**Table 5:** Classification table.

	Predicted		
Observed	0	1	% Correct
0	559	252	68.9
1	129	168	56.6

Note: The cut-off value is set to 0.3.

## Data Availability

All data are available from the corresponding author upon reasonable request.
